# Patient experience with bronchoscopy: topical versus monitored anesthesia

**DOI:** 10.1186/s12890-024-02954-6

**Published:** 2024-04-04

**Authors:** Chun-Ta Huang, Rou-Jun Chou, Geng-Ning Hu, Tien-Cheng Lee, Yi-Ju Tsai, Chao-Chi Ho

**Affiliations:** 1 Department of Internal Medicine, National Taiwan University Hospital, No. 7 Chung-Shan South Rd, Taipei 100, Taipei, Taiwan; 2 Graduate Institute of Clinical Medicine, National Taiwan University, Taipei, Taiwan; 3 Division of Respiratory Therapy and Chest Medicine, Department of Internal Medicine, Cathay General Hospital, Taipei, Taiwan; 4 Division of Pulmonary Medicine, Department of Internal Medicine, National Taiwan University Hospital, Hsin-Chu Branch, Hsinchu, Taiwan; 5 Department of Medicine, College of Medicine, National Taiwan University, Taipei, Taiwan; 6 School of Medicine, College of Medicine, Fu Jen Catholic University, New Taipei City, Taiwan

**Keywords:** Anesthesia, Bronchoscopy, Complication, Satisfaction, Visual analogue scale

## Abstract

**Background:**

This study aimed to compare patient experiences during bronchoscopy procedures using either topical anesthesia (TA) or monitored anesthesia care (MA). The goal was to identify circumstances where patients could achieve similar levels of tolerance and satisfaction using only TA, especially in resource-limited settings.

**Methods:**

This study included consecutive patients who underwent bronchoscopy with either TA or MA. Data collected included demographics, indications for bronchoscopy, procedure time, and complications during the procedure. A quality assurance survey was administered to assess patient experience and satisfaction with both procedures. A pre-specified subgroup analysis was performed based on procedure invasiveness and time.

**Results:**

This study enrolled 350 (TA 251; MA 99) patients, with an average age of 65 years. Main indications for bronchoscopy included tumor diagnosis (38%), esophageal cancer staging (18%), and pulmonary infection (17%). The average duration of the procedures was 20 min, with MA being associated with a significantly longer procedure time than TA (31 min vs. 16 min; *P* < 0.001). The overall satisfaction rating with bronchoscopy was significantly higher in the MA group (visual analogue scale, 8.9 vs. 8.2; *P* = 0.001). Subgroup analyses showed that when less invasive or shorter procedures were performed, TA patients reported tolerance and satisfaction levels comparable to MA patients.

**Conclusions:**

Bronchoscopy with MA offered patients a better experience and greater satisfaction; however, in settings with limited resources, TA alone may provide similar levels of patient tolerance and satisfaction during less invasive or shorter procedures.

**Supplementary Information:**

The online version contains supplementary material available at 10.1186/s12890-024-02954-6.

## Introduction

Since its inception in 1897, bronchoscopy has provided access to the lower airways in humans and has rapidly evolved to demonstrate its clinical utility [1]. In recent decades, we have seen remarkable progress in newer techniques such as endobronchial ultrasound, ultrathin bronchoscopes, electromagnetic navigation bronchoscopy, and virtual bronchoscopy, all of which have shown promising clinical applications [1]. In addition, interventional pulmonology has introduced a wide range of auxiliary diagnostic and therapeutic modalities, including cryobiopsy, airway stenting, thermoplasty, microwave, coils, and thermal vapor ablation [1, 2]. These advances have greatly improved the diagnosis and treatment of various disorders such as lung cancer, chronic obstructive pulmonary disease, asthma, and interstitial lung disease [2]. Today, bronchoscopy is an indispensable tool for pulmonologists.

Bronchoscopy is generally considered a safe procedure with a low complication rate [3]. However, it can cause anxiety, distress, and pain in patients. To alleviate these symptoms and improve patient tolerance, several society guidelines recommend the use of adequate sedation and topical anesthesia [4–6]. The practice of peri-bronchoscopy sedation varies across different settings and institutions due to factors such as limited resources for anesthesia and post-procedure monitoring, high patient volume, or patient preference [7, 8]. As a result, topical anesthesia (TA) alone or in combination with monitored anesthesia care (MA) or general anesthesia (GA) is commonly used for bronchoscopy worldwide [7, 9]. A recent study, for the first time, compared perioperative discomfort and patient satisfaction between patients undergoing bronchoscopy with TA or GA, and found that GA significantly reduced discomfort and improved patient satisfaction [10]. 

Although the use of sedative agents in GA can reduce anxiety and pain and induce antegrade amnesia in patients, it may also be associated with adverse effects such as hemodynamic instability, respiratory suppression, and additional costs [11]. It has been suggested that some patients undergoing relatively non-stimulating or short-duration procedures, such as inspection of the tracheobronchial tree, may be suitable for TA alone [12]. However, to date, there is little to no evidence in the literature to support or contribute to a discussion of this issue. Therefore, the current study aimed to investigate the procedures conducted during bronchoscopy, their details, and the experience of both patients and operators with the procedures under TA or MA.

## Methods

### Patients and study settings

This study was conducted at National Taiwan University Hospital, in Taipei, Taiwan. Consecutive patients who underwent bronchoscopy with either topical or monitored anesthesia between September 2022 and March 2023 were retrospectively identified for eligibility. Patients aged 18 years or older who had completed our quality assurance survey were included. Patients with a tracheostomy, cognitive impairment, or an incomplete survey were excluded from this study. This study has been carried out in accordance with the Declaration of Helsinki for experiments involving humans. The study protocol was approved by the Research Ethics Committee of National Taiwan University Hospital (202306094RIND), and the need for informed consent was waived due to the retrospective and non-interventional nature of the study.

### Bronchoscopy

All bronchoscopic procedures were performed by supervised pulmonology fellows under the guidance of attending pulmonologists. The choice of instruments and interventions was based on the clinical diagnosis and condition of each patient, and was at the discretion of the responsible pulmonologists.

For airway inspection, bronchial washing, bronchoalveolar lavage (BAL), endobronchial biopsy (EBB), and transbronchial biopsy (TBB), we utilized Olympus bronchoscopes including BF-1TQ290, BF-260, BF-Q290, and BF-P290 (Tokyo, Japan). Transbronchial needle aspiration (TBNA) was performed using Olympus BF-UC260FW equipped with a linear-probe endobronchial ultrasound (EBUS). Radial-probe EBUS (UM-S20-17 S; Olympus) was utilized for the localization of peripheral pulmonary lesions. The EBUS system was operated through the EVIS EUS Endoscopic Ultrasound Center (EU-ME2 PREMIER PLUS; Olympus).

During the study period, patients were given the option to undergo bronchoscopy under either MA plus TA or TA alone. TA was administered using local sprays of 2% lidocaine to the vocal cords, trachea, and carina, using a spray-as-you-go technique. MA was achieved through an intravenous injection. This included a combination of midazolam, alfentanil, and propofol, with the optional addition of ketamine and/or lidocaine. The specific combination was determined by the attending anesthesiologists. A bispectral index sensor (BIS; Medtronic, Minneapolis, MN, US) was routinely used to monitor the level of consciousness under intravenous anesthesia, with the sedation level targeted to achieve a BIS of 60–80 and a modified observer’s assessment of alertness and sedation (MOAA/S) score of 2–3 [13, 14]. 

### Data collection

Patient data collected in this study encompassed various aspects, including demographics, indications for bronchoscopy, procedure time (measured from insertion of the bronchoscope into the nasal or oral cavity to its removal), prior experience of the patients with bronchoscopy, types of bronchoscopic procedures performed, and complications encountered during the procedure. The recorded complications consisted of bleeding, O_2_ desaturation, and hemodynamic alteration. The severity of bleeding was assessed using the Nashville Bleeding Scale [15]. O_2_ desaturation was defined as a transcutaneous O_2_ saturation below 90% for a minimum duration of 5 s, regardless of the use of supplemental O_2_ [16]. Hemodynamic alteration was characterized by a systolic blood pressure decrease to below 90 mmHg or the occurrence of new brady- or tachy-arrhythmia [17]. 

During the study period, a quality assurance survey was administered to each patient, both before and after undergoing bronchoscopy, to assess their experience and satisfaction with the procedure. In addition, the operators were queried regarding their assessment of patient perception at the conclusion of the bronchoscopic procedure. To evaluate the survey items, a visual analogue scale (VAS) score ranging from 0 to 10 was employed. The detailed survey form can be found in Additional file 1.

### Statistical analysis

Descriptive statistics were presented as mean ± standard deviation for continuous variables, while count (%) was used for categorical variables. Between-group comparisons were conducted using appropriate statistical tests, such as χ2 or Fisher’s exact test for categorical variables, and the independent sample t-test for continuous variables, based on the distribution of the data.

Considering that the choice of anesthesia modality may vary among different patient groups based on expected procedure time and invasiveness, subgroup analyses were performed with patients undergoing more invasive procedures (EBB, EBUS-TBB, and TBNA) and those undergoing less invasive procedures (BAL, bronchial washing, and inspection), as well as with patients with longer (≥ 20 min) or shorter (< 20 min) procedure durations. The main focus of the subgroup analyses was on three patient-oriented outcomes: tolerance of the procedure, overall satisfaction rate, and willingness to undergo a re-examination.

Statistical analyses were conducted using version 20.0 of the SPSS software (IBM Corp.; Armonk, NY, US). A two-tailed *P* value less than 0.05 was considered statistically significant.

## Results

### Study subjects

**Fig. 1:**
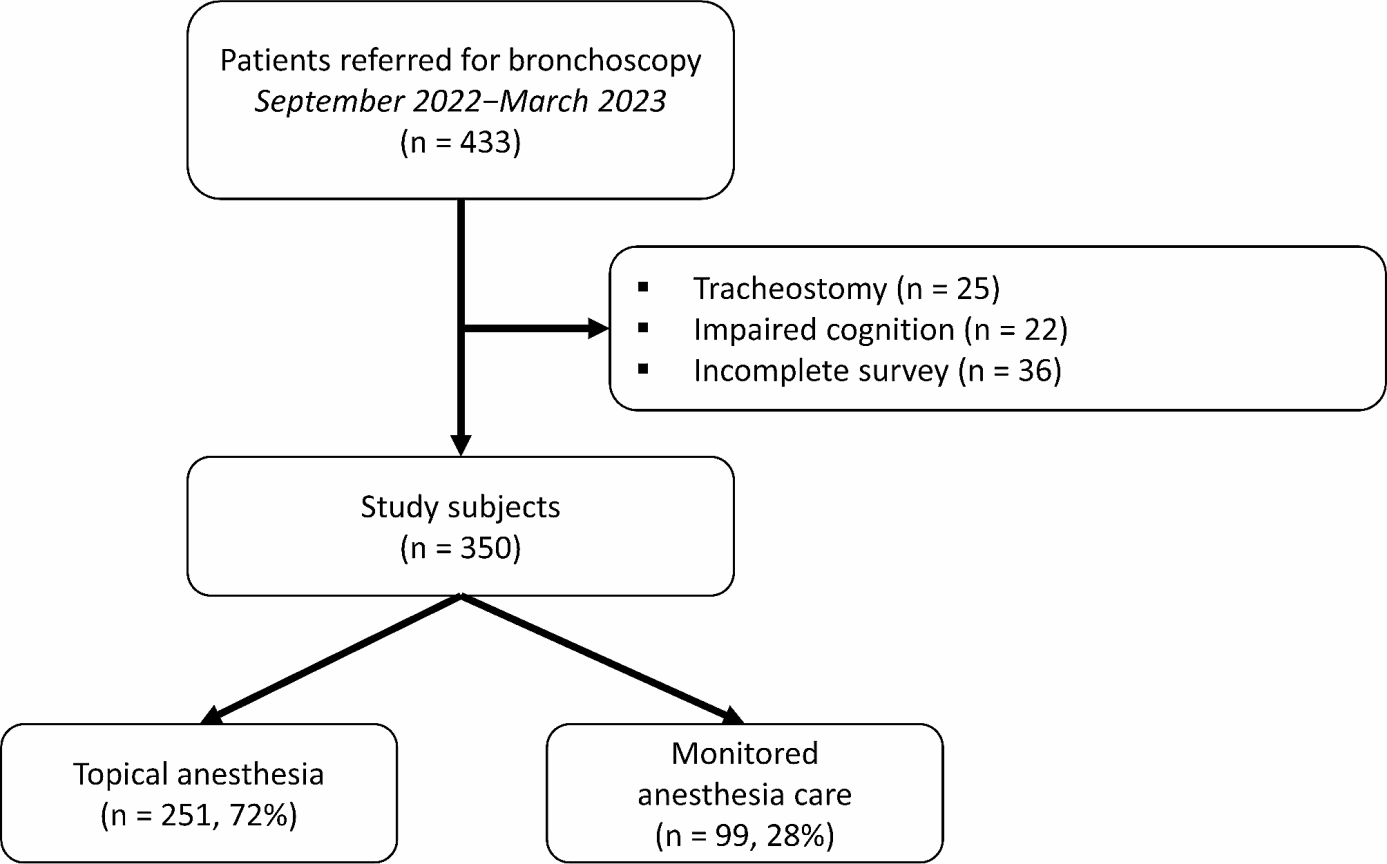
Study flow diagram

A total of 350 patients were enrolled in the study, as shown in Fig. 1. Among them, 251 (72%) received TA while 99 (28%) received MA. The average age of the study population was 65 years, and 212 (61%) of the patients were male, as summarized in Table 1. The main indications for bronchoscopy included tumor diagnosis (38%), esophageal cancer staging (18%), and pulmonary infection (17%). There were no significant differences observed in terms of age and gender distribution between patients receiving TA and those receiving MA. In the MA group, the predominant indications for bronchoscopy were tumor diagnosis (61%) and mediastinal lesion assessment (21%). TA patients, however, underwent bronchoscopy primarily for tumor diagnosis (29%), esophageal cancer staging (25%), and pulmonary infection (21%).

**Table 1:** Characteristics of study participants

	Total cohort	Topical anesthesia	Monitored anesthesia care	
Characteristic	*N* = 350	*N* = 251	*N* = 99	*P* value
Age, years	65 ± 13	64 ± 12	66 ± 14	0.134
≥65 years	202 (58)	142 (57)	60 (61)	0.492
Male sex	212 (61)	160 (64)	52 (53)	0.053
Indication				
Tumor diagnosis	132 (38)	72 (29)	60 (61)	< 0.001
Esophageal cancer staging	63 (18)	62 (25)	1 (1)	
Pulmonary infection	60 (17)	53 (21)	7 (7)	
Hemoptysis	32 (9)	26 (10)	6 (6)	
Mediastinal lesion	22 (6)	1 (1)	21 (21)	
Airway inspection	12 (3)	12 (5)	0 (0)	
Diffuse lung disease	9 (3)	7 (3)	2 (2)	
Atelectasis	8 (2)	7 (3)	1 (1)	
Tracheoesophageal fistula	6 (2)	5 (2)	1 (1)	
Chronic cough	3 (1)	3 (1)	0 (0)	
Others	3 (1)	3 (1)	0 (0)	

### Procedure details

A total of 249 patients (71%) underwent their first bronchoscopy, as shown in Table 2. The average duration of the procedures was 20 min, with MA patients having a significantly longer procedure time than TA patients (31 min vs. 16 min; *P* < 0.001). The choice of main procedures differed between the TA and MA groups. EBUS-TBB (43%) and TBNA (29%) were predominantly performed under MA, while inspection (34%) and bronchial washing (31%) were more commonly carried out under TA. MA patients were more likely to undergo more invasive procedures, including EBB, EBUS-TBB, and TBNA, compared to TA patients (80% vs. 34%; *P* < 0.001). Regarding complications, grade 2 or higher bleeding was more frequently observed in the MA group than in the TA group (13% vs. 6%; *P* = 0.026). Occurrences of O_2_ desaturation (< 1%) and hemodynamic changes (< 1%) were uncommon. No procedure-related mortality was encountered throughout the study period.

**Table 2:** Details of bronchoscopy

	Total cohort	Topical anesthesia	Monitored anesthesia care	
Characteristic	*N* = 350	*N* = 251	*N* = 99	*P* value
First-ever experience	249 (71)	173 (69)	76 (77)	0.145
Procedure time, min	20 ± 14	16 ± 13	31 ± 13	< 0.001
≥20 min	159 (45)	82 (33)	77 (78)	< 0.001
Main procedure				
EBUS-TBB	112 (32)	69 (28)	43 (43)	< 0.001
Inspection	89 (25)	84 (34)	5 (5)	
Bronchial washing	87 (25)	77 (31)	10 (10)	
TBNA	30 (9)	1 (1)	29 (29)	
EBB	19 (5)	14 (6)	5 (5)	
BAL	11 (3)	6 (2)	5 (5)	
EBUS-TBB + TBNA	2 (1)	0 (0)	2 (2)	
Procedure type				
Less invasive^a^	187 (53)	167 (67)	20 (20)	< 0.001
More invasive^b^	163 (47)	84 (34)	79 (80)	
Complication				
Grade 2 or higher bleeding	28 (8)	15 (6)	13 (13)	0.026
O_2_ desaturation	3 (1)	2 (0.8)	1 (1)	0.845
Hemodynamic change	1 (1)	1 (1)	0 (0)	0.999

^a^ Including BAL, bronchial washing, and inspection

^b^ Including EBB, EBUS-TBB, and TBNA

EBB, endobronchial biopsy; EBUS-TBB, endobronchial ultrasound-guided transbronchial biopsy; TBNA, transbronchial needle aspiration; BAL, bronchoalveolar lavage

### Patient and operator experience

Before bronchoscopy, patients’ anxiety levels and understanding of the anesthesia were comparable between the TA and MA groups (Table 3). However, TA patients reported experiencing more discomfort from oropharyngeal anesthesia and during the bronchoscopy procedure, along with more severe cough during the procedure, than MA patients (VAS 4.6 vs. 3.0, 5.4 vs. 2.1, and 4.1 vs. 1.9, respectively; all *P* < 0.001). As expected, MA patients had a lower likelihood of recalling the details of the procedure compared to TA patients (VAS 1.6 vs. 8.8; *P* < 0.001).

**Table 3:** Patient and operator experience with bronchoscopy

	Total cohort	Topical anesthesia	Monitored anesthesia care	
Variable	*N* = 350	*N* = 251	*N* = 99	*P* value
Evaluated by the patient				
Before bronchoscopy				
Understanding about the type of anesthesia	4.2 ± 3.9	4.2 ± 4.0	4.1 ± 3.8	0.864
Anxiety before bronchoscopy	4.5 ± 3.0	4.6 ± 3.1	4.4 ± 2.8	0.533
After bronchoscopy				
Discomfort from oropharyngeal anesthesia	4.2 ± 2.9	4.6 ± 3.0	3.0 ± 2.2	< 0.001
Discomfort during bronchoscopy	4.4 ± 3.0	5.4 ± 2.9	2.1 ± 2.0	< 0.001
Cough during the procedure	3.5 ± 2.9	4.1 ± 2.9	1.9 ± 2.2	< 0.001
Recalling the details of the procedure	6.8 ± 4.0	8.8 ± 2.1	1.6 ± 2.6	< 0.001
Post-procedural throat discomfort	3.5 ± 2.7	3.6 ± 2.8	3.2 ± 2.3	0.226
Tolerance of the procedure	7.4 ± 2.6	7.4 ± 2.6	7.5 ± 2.7	0.615
Overall rate of satisfaction	8.4 ± 1.9	8.2 ± 1.9	8.9 ± 1.6	0.001
Consent to a re-examination	7.3 ± 3.1	7.1 ± 3.3	7.7 ± 2.5	0.059
Evaluated by the operator				
Patient discomfort	3.7 ± 2.5	4.3 ± 2.5	2.3 ± 2.0	< 0.001
Cough during bronchoscopy	3.2 ± 2.6	3.6 ± 2.5	2.2 ± 2.3	< 0.001
Procedural interference by cough	2.8 ± 2.6	3.1 ± 2.6	2.0 ± 2.3	< 0.001

The overall satisfaction rate with bronchoscopy was significantly higher in the MA group than the TA group (VAS 8.9 vs. 8.2; *P* = 0.001). Furthermore, there was a trend suggesting that MA patients were more inclined to undergo repeat bronchoscopy than TA patients (VAS 7.7 vs. 7.1; *P* = 0.059).

From the operators’ perspective, patients experienced less discomfort and cough during bronchoscopy when placed under MA compared to TA (VAS 3.3 vs. 4.3, 2.2 vs. 3.6, respectively; both *P* < 0.001). In addition, the operators reported that cough interfered less with the procedure in the MA group than the TA group (VAS 2.0 vs. 3.1; *P* < 0.001).

### Subgroup analyses

In the subgroup analyses involving patients who underwent less invasive procedures or had a procedure time of less than 20 min, no significant differences were found in terms of procedure tolerance, overall satisfaction, and willingness to repeat bronchoscopy (Table 4). However, when more invasive procedures were performed during bronchoscopy, MA patients reported significantly higher overall satisfaction ratings (VAS 8.9 vs. 8.0; *P* = 0.002) and were more willing to undergo re-examination (VAS 7.7 vs. 6.7; *P* = 0.034) than TA patients. Similar findings were observed among patients with a procedure duration of 20 min or longer.

**Table 4:** Subgroup analyses

	Topical anesthesia	Monitored anesthesia care	*P* value
**Procedure invasiveness**			
*More invasive (EBB, EBUS-TBB, and TBNA)*			
Tolerance of the procedure	7.1 ± 2.8	7.8 ± 2.3	0.099
Overall rate of satisfaction	8.0 ± 2.0	8.9 ± 1.5	0.002
Consent to a re-examination	6.7 ± 3.5	7.7 ± 2.6	0.034
*Less invasive (BAL, bronchial washing, and inspection)*			
Tolerance of the procedure	7.5 ± 2.5	6.5 ± 3.7	0.249
Overall rate of satisfaction	8.3 ± 1.9	8.9 ± 1.7	0.206
Consent to a re-examination	7.2 ± 3.2	7.9 ± 2.1	0.337
**Procedure time**			
*≥20 min*			
Tolerance of the procedure	7.0 ± 2.9	7.7 ± 2.5	0.090
Overall rate of satisfaction	7.8 ± 2.1	8.8 ± 1.6	0.001
Consent to a re-examination	6.6 ± 3.6	7.9 ± 2.5	0.008
*<20 min*			
Tolerance of the procedure	7.6 ± 2.5	7.0 ± 3.2	0.287
Overall rate of satisfaction	8.4 ± 1.9	9.1 ± 1.5	0.131
Consent to a re-examination	7.4 ± 3.1	7.1 ± 2.3	0.724

EBB, endobronchial biopsy; EBUS-TBB, endobronchial ultrasound-guided transbronchial biopsy; TBNA, transbronchial needle aspiration; BAL, bronchoalveolar lavage

## Discussion

As far as we know, this study is the first to assess the indications and details of bronchoscopy performed under TA and MA, as well as the differences in patient and operator experience. Moreover, this study highlights the circumstances under which patients can undergo bronchoscopy using TA only, with acceptable levels of tolerance and satisfaction. Our study found that bronchoscopy performed under MA resulted in less coughing and discomfort, as reported by both patients and operators, compared to TA. Patient satisfaction with the procedure was higher under MA than TA, and the procedure was less likely to be interrupted by coughing when performed under MA. Subgroup analyses showed that for less invasive or shorter bronchoscopies, patient tolerance, satisfaction, and willingness to repeat the examination were similar between those performed under MA and those under TA. In summary, our study indicates that bronchoscopy performed under MA provides greater comfort and satisfaction for patients than TA. However, for less invasive or shorter procedures, patients may still be suitable candidates for bronchoscopy using TA alone.

Although it may seem intuitive that GA or MA would be superior to TA in terms of patient comfort and tolerance during bronchoscopy, it was not until recently that Feng et al. [10] confirmed this idea through a head-to-head study between GA and TA. Our study builds on this knowledge by demonstrating that MA also reduced discomfort and improved patient satisfaction during bronchoscopy compared to TA. Furthermore, our analysis of procedure details revealed that MA was more likely to be used for patients undergoing more invasive or longer bronchoscopic procedures than TA. This finding is reasonable, as physicians and patients may prefer MA over TA for anticipated difficult or prolonged procedures, and vice versa. This suggests that while GA or MA for bronchoscopy is undoubtedly associated with improved patient well-being and satisfaction compared to TA, the choice of anesthesia modality in clinical practice is often conditional, depending not only on physician and patient preferences but also on the planned procedures.

The most notable aspect of this study is that, for the first time, we investigated the characteristics that can help identify patients who are suitable for bronchoscopy under TA while maintaining patient comfort. Using TA alone in bronchoscopy, the complications associated with GA or MA can be avoided, the time patients spend in the hospital can be reduced, and immediate discussion of results with the patients can be enabled [18]. We found that patients experienced similar levels of tolerance and satisfaction with bronchoscopy when undergoing less invasive procedures or when their procedure time was less than 20 min. These results are clinically plausible and practical, providing evidence to support shared decision-making regarding the choice of anesthesia for bronchoscopic procedures. Our findings are also consistent with previous statements that diagnostic bronchoscopy is typically well-tolerated without the use of sedation [19–21], and partially confirm our clinical observations as described above. Taken together, TA alone is a viable option for less invasive or shorter bronchoscopies for patients in resource-limited settings.

The incidence of complications during bronchoscopy and resulting morbidity is low [17, 22], with equivalent safety observed for bronchoscopy with and without moderate sedation [23]. Consistent with previous studies, our study did not encounter any complications with significant sequelae, and no procedure-related mortality was observed. Although grade 2 or higher bleeding was more common in the MA group than in the TA group, this may be explained by the difference in procedure types performed, with more invasive procedures being conducted in the MA group. The instances of O_2_ desaturation and hemodynamic changes were infrequent in both the MA and TA groups of our study population.

Advances in technology and in techniques have increased the capabilities of bronchoscopy, but have also made it a more complex and time-consuming procedure [2]. Our study aimed to assist with resource allocation in certain clinical settings, rather than argue against the use of sedation during bronchoscopy [4–6]. It is important to note that while TA can reduce sensation and cough reflex in the oropharynx, larynx, and major airways, it alone cannot fully alleviate patient anxiety, enhance cooperation, or prevent movement during the procedure. The interactions between the operator, nurse, and patient can also impact the patient’s experience with bronchoscopy. However, this issue was not explored in our study or in the existing literature, and may warrant further investigation.

A couple of limitations relevant to this study should be discussed herein. Firstly, this was not a randomized controlled trial, and therefore may be subject to some biases inherent to observational studies. However, due to reimbursement limitations within our healthcare insurance system, the use of MA for bronchoscopy is left to the discretion of the patient, and is an out-of-pocket expense. In addition, limited manpower and capacity for MA present challenges in conducting such a study. As a result, while not impossible, conducting a clinical trial on this topic may be challenging. Secondly, this study was conducted in a tertiary care referral center, and the applicability of our findings to other institutions may be uncertain. However, we believe that our most notable finding - that patient experiences with MA and TA are comparable for less invasive or shorter bronchoscopic procedures - should be relevant to other settings. This is because these types of procedures are more commonly performed and are more familiar to bronchoscopists nationwide. Lastly, although our study suggests that TA may be applicable for bronchoscopy under certain circumstances, it should be borne in mind that major society guidelines recommend sedation for all patients undergoing bronchoscopy, provided there are no contraindications [5, 6]. 

## Conclusions

In summary, while bronchoscopy with MA undoubtedly provides patients with a better experience and more satisfaction, in resource-limited settings, TA alone may be comparable to MA in terms of patient tolerance, satisfaction, and willingness to undergo repeat examinations when less invasive or shorter procedures are performed.

### Electronic supplementary material

Below is the link to the electronic supplementary material.

Supplementary Material 1

## Data Availability

The datasets used and/or analyzed during the current study are available from the corresponding author on reasonable request.

## Abbreviations

BAL: bronchoalveolar lavage
EBB: endobronchial biopsy
EBUS: endobronchial ultrasound
GA: general anesthesia
MA: monitored anesthesia care
TA: topical anesthesia
TBB: transbronchial biopsy
TBNA: transbronchial needle aspiration
VAS: visual analogue scale
