# Supplementary material for: Patient experience with bronchoscopy: topical versus monitored anesthesia
Source: BMC Pulm Med. 2024 Apr 4;24:164. doi: 10.1186/s12890-024-02954-6 (PMC10996097; doi:10.1186/s12890-024-02954-6)
Supplement: Supplementary file 1 — Supplementary Material 1 [file 12890_2024_2954_MOESM1_ESM.docx]

**Survey form**

**Please circle the most appropriate statement relative to your experience with bronchoscopy**

**Patient**

| **Before bronchoscopy** | | | |
| --- | --- | --- | --- |
| Understanding about the type of anesthesia | None | 0 1 2 3 4 5 6 7 8 9 10 | All |
| Anxiety before bronchoscopy | None | 0 1 2 3 4 5 6 7 8 9 10 | Worst |
| **After bronchoscopy** | | | |
| Discomfort from oropharyngeal anesthesia | None | 0 1 2 3 4 5 6 7 8 9 10 | Worst |
| Discomfort during bronchoscopy | None | 0 1 2 3 4 5 6 7 8 9 10 | Worst |
| Cough during the procedure | None | 0 1 2 3 4 5 6 7 8 9 10 | Worst |
| Recalling the details of the procedure | None | 0 1 2 3 4 5 6 7 8 9 10 | All |
| Tolerance of the procedure | Zero | 0 1 2 3 4 5 6 7 8 9 10 | Excellent |
| Overall rate of satisfaction | Zero | 0 1 2 3 4 5 6 7 8 9 10 | 100% |
| Consent to a re-examination | Absolutely unwilling | 0 1 2 3 4 5 6 7 8 9 10 | Fully willing |
| Post-procedural throat discomfort | None | 0 1 2 3 4 5 6 7 8 9 10 | Worst |

**Survey form**

**Please circle the most appropriate statement relative to your experience with bronchoscopy**

**Operator**

| **After bronchoscopy** | | | |
| --- | --- | --- | --- |
| Patient discomfort | None | 0 1 2 3 4 5 6 7 8 9 10 | Worst |
| Patient coughing during bronchoscopy | None | 0 1 2 3 4 5 6 7 8 9 10 | Worst |
| Procedural interference due to coughing | None | 0 1 2 3 4 5 6 7 8 9 10 | Worst |
